# *Staphylococcus aureus* induces COX-2-dependent proliferation and malignant transformation in oral keratinocytes

**DOI:** 10.1080/20002297.2019.1643205

**Published:** 2019-07-22

**Authors:** Yuxia Wang, Shiyu Liu, Bolei Li, Yaling Jiang, Xinxuan Zhou, Jing Chen, Mingyun Li, Biao Ren, Xian Peng, Xuedong Zhou, Lei Cheng

**Affiliations:** a State Key Laboratory of Oral Diseases & National Clinical Research Center for Oral Diseases, Sichuan University, Chengdu, China; b Department of Cariology and Endodontics, West China Hospital of Stomatology, Sichuan University, Chengdu, China; c Department of Cariology and Endodontics, Hospital of Stomatology, Nankai University, Tianjin, China

**Keywords:** COX-2, Staphylococcus aureus, oral keratinocytes, proliferation, malignant transformation

## Abstract

The COX-2/PGE_2_ axis can play roles in mediating the progression of tumor. COX-2 induction was observed in oral cancer. In our previous study, we found *Staphylococcus aureus*, a pathogen prevalent in oral cancer, can activate the COX-2/PGE_2_ pathway in human oral keratinocyte (HOK) cells. Here, we investigated the proliferation of HOK cells affected by COX-2 induction and the role of COX-2 induction in the malignant transformation of HOK cells. We found *S. aureus* was able to facilitate HOK cell proliferation through upregulating COX-2 expression. With the induction of COX-2, expression of oral cancer-associated genes *cyclin D1* was upregulated and *p16* was downregulated. Transcriptome analysis showed that the “NF−kappa B signaling pathway” and “TNF signaling pathway” had the highest enrichment of differentially expressed genes (DEGs) with COX-2 over-expression. Seven upregulated genes (*jun*, *tlr4*, *cxcl1*, *lif*, *cxcl3*,* tnfrsf1β*, and *il1β*) in these two pathways were critical for the increased proliferation of HOK cells and might be associated with COX-2. Malignant transformation of cells was evaluated by soft agar colony formation assay and *S. aureus* infection promoted HOK cell colony formation. These results suggest the potential of *S. aureus* to induce the infection-associated malignant transformation of oral epitheliums through COX-2 activation.

## Graphical Abstract

**Figure:**
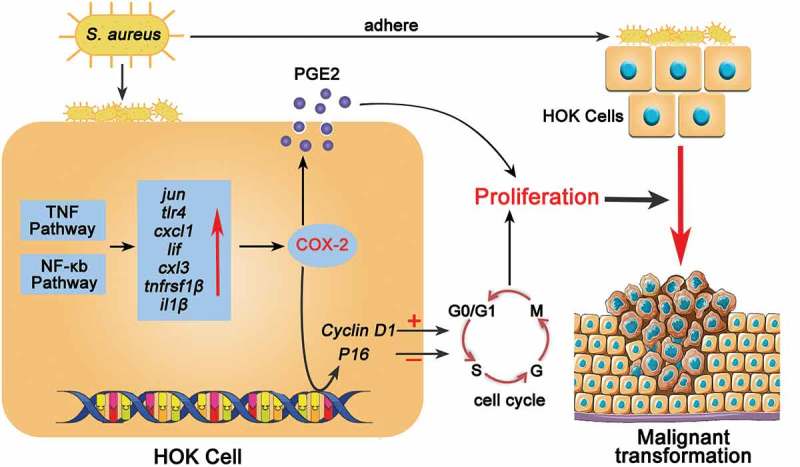

*Staphylococcus aureus* has long been recognized as a major human opportunistic pathogen that is involved in a variety of infectious diseases. In the oral tract, *S. aureus* can be isolated from saliva, the tongue, mucosal surfaces, supragingival tooth surfaces and periodontal pockets [1]. Soft tissue infection and jaw osteomyelitis in the oral and maxillofacial regions can be caused by this bacterium [2,3]. Moreover, *S. aureus* may play a role in dental implant failure [4,5]. Owing to its resistance to many kinds of antibiotics, *S. aureus* infection has been a large healthcare burden worldwide [6,7]. Recently, it has been suggested that *S. aureus* may also be associated with oral cancer, the eighth most prevalent cancer in the world [8]. *S. aureus* is reported to be frequently detected in oral cancer patients’ oral cavities [9,10]. However, whether *S. aureus* participates in oral cancer development remains unclear.

Cancer is a leading cause of death and confers an enormous burden on society. Accumulated evidence supports the point that inflammatory states promote the initiation and growth of some tumours [11,12]. The contribution of microorganisms to inflammation-induced cancer arising from infections has been firmly established in recent years [13,14]. *S. aureus* is a major pathogen that causes severe inflammation at many sites, including the oral mucosa, skin, bone, blood and intestines [15–18]. Whether *S. aureus* promotes oral cancer development through the induction of inflammation is a pertinent question.

In our previous study, we found that *S. aureus* can induce Cyclooxygenase-2 (COX-2) expression and PGE_2_ production in human oral keratinocyte (HOK) cells [19]. COX-2 is an enzyme that mediates the synthesis of prostaglandins (PGE_2_, PGD_2_, PGF2α, PGI_2_, and thromboxane) and plays an important role in the inflammatory response [20]. Among the five prostaglandins, PGE_2_ has been shown to be involved in carcinogenesis due to its functions in inducing cell proliferation, invasion, metastasis and angiogenesis [21,22]. It has been demonstrated that the COX-2/PGE_2_ pathway plays key roles in mediating the hallmarks of cancer and aids tumour progression [23]. COX-2 induction in the oral tract, was observed in oral cancer patients as well as in cancer tissues and was suggested to be related to head and neck cancer through regulating tumour-associated factors such as VEGF [24–26]. A study by Shibata [27] pointed out that COX-2 expression was correlated with the grade of oral dysplasia, and higher expression of COX-2 in severe dysplastic lesions than in mild and moderate dysplastic lesions was observed, verifying that COX-2 may be involved in the regulation of cell proliferation in the progression from normal mucosa to squamous cell carcinoma [28]. COX-2 also acts as a critical mediator of the metastatic activity of oral cancer cells in the tumour microenvironment; overexpression of COX-2 enhanced cell migration in oral cancer cells [29]. Therefore, COX-2 inhibitors are already in clinical trials for the prevention of oral cancer [30].

Considering the important role of COX-2 in oral cancers, it would be of great interest to study the potential for the malignant transformation of oral epithelial cells after the induction of COX-2 by *S. aureus*. The concept that the induction of cell proliferation by infection might increase the incidence of cell transformation and the rate of tumour development has been established [31]. Thus in the present study, we tested the proliferation of HOK cells after *S. aureus* stimulation. We found overexpression of COX-2 by *S. aureus* infection facilitated HOK proliferation but independent of PGE_2_ production. COX-2 induction regulated the oral cancer-associated genes *cyclin D1* and *p16*. The ‘NF−kappa B signaling pathway’ and ‘TNF signaling pathway’ may form candidate mechanisms for the promotive effect of COX-2 on HOK cell proliferation. Seven upregulated genes (*jun, tlr4, cxcl1, lif, cxcl3, tnfrsf1β, il1β*) in these two pathways were critical for the increased proliferation rate of HOK cells. In addition, *S. aureus* infection promoted malignant transformation in HOK cells.

## Materials and methods

### Cell lines, bacterial strains and culture

The commercial human oral keratinocyte (HOK) cell line was cultured in high glucose Dulbecco’s modified Eagle’s medium (DMEM, HyClone, Logan, UT) containing 10% foetal bovine serum (FBS, Gibco, Thermo Fisher Scientific, Inc., Waltham, MA) and 1% penicillin–streptomycin antibiotic mixture (PS, HyClone, Logan, UT). The cells were cultured in an incubator with 5% CO_2_ and 95% air at 37°C. *S. aureus* strain ATCC 25,923 was routinely cultured in tryptone soya broth (TSB, Oxoid, Basingstoke, UK), and 1.5% agar was added when needed.

### S. aureus inactivation

Overnight cultures of *S. aureus* were centrifuged at 4,000 rpm for 15 min and then washed and resuspended in PBS. The suspension was diluted 1:50 with fresh DMEM containing 10% FBS and incubated at 37°C for growth to the exponential phase. *S. aureus* cells in the exponential phase were centrifuged. The supernatants were filtered through a 0.22-μm microfiltration membrane. The pellets were washed with PBS and suspended in DMEM. Alternatively, the pellets were washed and suspended in sterile PBS and were then heat-inactivated at 80°C for 20 min in a water bath, followed by harvesting and suspension in DMEM.

### Infection assay

HOK cells were incubated in 6-well plates to 80% confluence. The supernatants were removed, and the cells were washed twice with PBS. Then, cells were infected at an MOI of 100:1 with bacterial cells suspended in DMEM or treated with the filtered supernatants at a proportion of 10% (v/v) and incubated at 37°C for 45 min. After being washed with PBS, the cells were lysed with TRIzol reagent (Invitrogen, CA) and stored at −80°C for RNA extraction and real-time quantitative PCR. Alternatively, HOK cells were incubated in 6-well plates to 80% confluence with either 0.025% dimethyl sulfoxide (DMSO) or 20 μM NS-398 (Sigma-Aldrich, Saint Louis, MI), a specific COX-2 inhibitor, dissolved in DMSO at an optimal dose. Then, the cells were infected with bacterial cells suspended in DMEM and incubated at 37°C for 45 min. Wells with bacterial cells only were used as the negative control, and DMEM was used as the blank control. The supernatants were centrifuged at 2,000 rpm for 25 min to remove the cells, and the liquid supernatants were stored at −80°C for ELISA. After being washed with PBS, cells were lysed with TRIzol reagent and either stored at −80°C for RNA extraction or, were harvested by trypsin digestion and centrifugation at 1,000 rpm for 5 min and were washed with PBS, with the pellets stored in liquid nitrogen for Western blotting.

### RNA extraction and quantitative real-time PCR

To quantify COX-2 mRNA expression, total RNA was isolated following the instructions provided with TRIzol reagent (Invitrogen, CA). The total RNA yield and purity were determined by measuring the absorbance at 260 nm and 280 nm using a NanoDrop 2000 spectrophotometer (Thermo Fisher Scientific Inc., Waltham, MA). cDNA was then synthesized using a PrimeScript RT reagent kit with gDNA Eraser (Takara Clontech, Japan) according to the manufacturer’s instructions. Real-time PCR was performed on a C1000 Touch™ thermal cycler instrument (Bio-Rad, Philadelphia, PA) with SYBR reagent (Takara, Dalian, China) following the manufacturer’s instructions. Amplification was performed according to the reported protocol with some modifications [32]. A 25-µL mixture of 12.5 µL SYBR qPCR Mix (Takara, Dalian, China), 2 µL PCR primers [33] mix (10 µM), 2 µL diluted template cDNA, and 8.5 µL deionized distilled water was processed for RT-PCR. The relative fold changes in COX-2 expression were normalized to glyceraldehyde-3-phosphate dehydrogenase (GAPDH) expression. The PCR primers used in this study are listed in Supplementary Table 1.

### Western blotting

Frozen cells were lysed in RIPA buffer (1% Triton X-100; 1% sodium deoxycholate; 0.1% SDS; 150 mM NaCl; 50 mM Tris-HCl, pH 7.8; and 1 mM EDTA) containing protease inhibitor cocktail (Roche, Mannheim, Germany) for 30 min on ice and were then removed by centrifuging at 12,000 × g for 5 min. Proteins were quantified by the BCA method, separated by SDS-PAGE, transferred to nitrocellulose membranes, and analyzed with Western blotting. The primary and secondary antibodies used included mouse anti-total actin (Sigma-Aldrich, St. Louis, MO), mouse anti-COX-2 and HRP-conjugated anti-mouse polyclonal antibodies (Santa Cruz Biotechnologies).

### PGE_2_ ELISA

The supernatant was collected for PGE_2_ measurement as described above. Briefly, HOK cells were cocultured with *S. aureus* at an MOI of 100:1 in DMEM without FBS for 45 min. The supernatants were harvested by centrifugation at 2,000 rpm for 25 min and filtered through a 0.22-μm microfiltration membrane. PGE_2_ production was measured by ELISA (Cayman Chemical, Ann Arbor, MI) according to the manufacturer’s instructions, with each sample measured in triplicate [34].

### Proliferation assay

In a 96-well plate, 2 × 10^3^ cells were incubated overnight for adherence. The supernatants were removed and, after washing with PBS, cells were measured using a CCK-8 assay kit (Dojindo) following the manufacturer’s instructions. The CCK-8 solution was diluted 1:10 with DMEM, and 100 µL of diluted solution was added into a well. After incubation for 2 h, the absorbance was measured at 450 nm using a microplate reader (Thermo Fisher Scientific Inc., Waltham, MA). In addition, other cells were treated with PGE_2_ (Sigma) at a concentration of 500 pg/mL, with heat-inactivated *S. aureus* at an MOI of 100:1, or with supernatants harvested from *S. aureus* culture at a proportion of 10% (v/v). Cells without any stimulation treatment were used as the negative control, and wells with no seeded cells but containing equal volumes of DMEM, inactivated *S. aureus* or supernatants were used as blank controls. The treated cells were incubated and measured using the CCK-8 assay kit (Dojindo) as described above. The OD values of each well were measured to represent the proliferation of HOK cells. All experiments were performed at least in triplicate.

### Soft agar colony formation assay

In a 6-well plate, 7.5 × 10^5^ HOK cells with or without heat-inactivated *S. aureus* at an MOI of 100:1 were resuspended in DMEM containing 0.3% agar and 10% FBS and layered on a base agar consisting of DMEM supplemented with 0.6% agar and 10% FBS. Following agar solidification, growth medium was added to the cell agar layer. Twelve days later, colonies were imaged under a microscope, and the size of each colony in three random images was measured and t test was used for statistical analysis.

### Small-interfering RNA transfection assay

HOK cells were plated at a seeding density of 2 × 10^5^ per well in 6-well plates and grown to 60–80% confluence at 37°C in cell growth medium without antibiotics in a CO_2_ incubator. The cells were then transfected either with 60 pmol human COX-2 siRNA or with 60 pmol control siRNA containing a scrambled sequence that would not lead to the specific degradation of any known cellular mRNA (Santa Cruz Biotechnology, Santa Cruz, CA). After a 6-h incubation at 37°C in a CO_2_ incubator, the transfection mixture was removed, and normal growth medium was added. The cells were incubated for 18 h and were then harvested. Then, 96-well plates were seeded with the harvested cells at a density of 2 × 10^5^ cells per well, and the infection and proliferation assays were performed with the protocols described above.

### Generation of the COX-2 overexpression cell line

The lentiviral vectors LV-PTGS2 and CON238 were generated by Genechem Co., Ltd., Shanghai, China. LV-PTGS2 encodes the target COX-2 gene, and CON238 was used as the control vector. HOK cells were seeded in 6-well plates and infected with either LV-PTGS2 or CON238 at an MOI of 5:1 by enhanced infection solution containing polybrene (5 μg/mL). The cells were cultured in DMEM containing 10% FBS, and 48 h after infection, puromycin was added to the medium at a concentration of 2.0 μg/mL. Cells with a transfection efficiency of greater than 70% were used for experiments after confirmation. The HOK cell line variant with COX-2 overexpression was named OE, and the control variant was named NC.

### Transcriptome analysis by RNA-sequencing

RNA of six samples (NC triplicates and OE triplicates) was isolated using TRIzol reagent (Invitrogen Life Technologies) according to the manufacturer’s instructions. The quality and concentration of the RNA were determined using a NanoDrop spectrophotometer (Thermo Scientific), and the integrity was confirmed by a Bioanalyzer 2100 system (Agilent). mRNA was purified from the high-quality total RNA using poly-T oligo-attached magnetic beads. Fragmentation was carried out using divalent cations under elevated temperatures in an Illumina proprietary fragmentation buﬀer. First-strand cDNA was synthesized using random oligonucleotides and SuperScript II; second-strand cDNA was subsequently synthesized using DNA polymerase I and RNase H. Remaining overhangs were converted into blunt ends via the exonuclease/polymerase activities, and the enzymes were then removed. After adenylation of the 3ʹ ends of the DNA fragments, Illumina PE adapter oligonucleotides were ligated to the fragments to prepare for hybridization. To select cDNA fragments of the preferred length of 200 bp, the library fragments were purified using the AMPure XP system (Beckman Coulter, Beverly, CA). DNA fragments with ligated adaptor molecules on both ends were selectively enriched using Illumina PCR Primer Cocktail via a 15-cycle PCR. The products were purified (AMPure XP system) and quantified using the Agilent high-sensitivity DNA assay on the Bioanalyzer 2100 system (Agilent). The sequencing library was then sequenced on a HiSeq platform (Illumina) by Shanghai Personal Biotechnology Co., Ltd. [35,36].

Raw data were collated and estimated by calculating the total reads and bases, Q20 (the number of bases with an error rate of ≤ 1%), Q30 (the number of bases with an error rate of ≤ 0.1%), GC content and sequence duplication level. Then, low-quality reads were filtered, and reads containing adapters or poly-N were removed to obtain clean data, which were used for all downstream analyses. The reference genome Homo_sapiens GRCh38.dna.primary_assembly.fa was built by the Ensembl database (version 87.38) (http://www.ensembl.org/) and annotated based on the NCBI, UniProtKB (UniProt Knowledgebase, http://www.UniProt.org/help/uniprotkb), GO (Gene Ontology, http://geneontology.org/), EC (Enzyme Commission，http://enzyme.expasy.org/), EggNOG (Evolutionary Genealogy of Genes: Non-supervised Orthologous Groups; http://eggnog.embl.de/version_3.0/), and KEGG (Kyoto Encyclopedia of Genes and Genomes, http://ggp/) databases. An index of the reference genome was built using Bowtie v2.0.6 [37], and all trimmed reads were aligned to the reference genome using TopHat v2.0.4 [38].

The read counts mapped to each transcript were calculated using HTSeq 0.6.1p2 (http://www-huber.embl.de/users/anders/HTSeq). These data were normalized by RPKM (Reads per kilobases per million reads), and the results were used for the analysis of differentially expressed genes (DEGs). All DEGs were identified using the DESeq R package (1.18.0) with a cutoff of |fold change| > 2 and a threshold *p* value of < 0.05. GO enrichment analysis of DEGs was implemented by the GO Term Finder. Then, we used KAAS software to test the statistical enrichment of differentially expressed genes in KEGG pathways. The raw transcriptome reads reported in this paper have been deposited in the NCBI Sequence Read Archive with accessionnumber PRJNA428623.

### High-content screening assay

Virus skeleton plasmid H1 (312 ng), virus skeleton plasmid H2 (258 ng), and a plasmid (628 ng) containing targeting shRNA were transfected into 293 T cells with X-tremeGENE HP (2 μL, Roche). Forty-eight h later, 80 μL of supernatants containing targeting shRNA lentivirus was collected and added to HOK cells seeded at a density of 1,500 cells/well in a 96-well black-bottom plate. On the second day, cells with green fluorescence were imaged and counted by the cell cytometry system Celigo™ once daily [39]. Cell growth was observed continuously for 5 days, and cell growth curves were generated.

## Statistical analysis

Except for RNA-seq, all experiments were independently repeated three times. Comparisons between groups were analyzed by Mann-Whitney U test when n = 3, and analysis of variance (ANOVA) when n > 3, unless otherwise stated. The data are presented as the means ± SEs, and the results were considered to be statistically significant at *p* < 0.05.

## Results

### S. aureus activates the COX-2/PGE_2_ pathway in HOK cells

**Figure 1.**
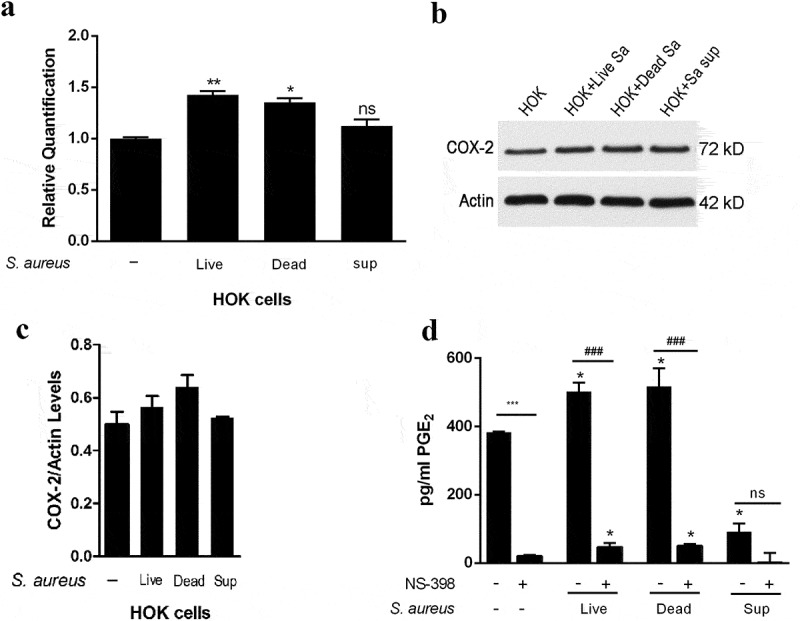
*S. aureus* activates the COX-2/PGE2 pathway in HOK cells. (a) Fold changes of COX-2 mRNA expression in HOK cells treated with live *S. aureus*, heat-inactivated *S. aureus*, or *S. aureus* supernatants (n = 3). (b and c) Quantification of COX-2 protein expression by Western blotting (n = 3). (d) The quantity of PGE_2_ secretion measured by ELISA. NS-398, a small molecule specific inhibitor of COX-2 (n = 3). The data are presented as the means ± standard errors, and the asterisks represent significant differences compared with the non-treated group (^＊^ *p* < 0.05, ^＊＊^*p* < 0.01, ^＊＊＊^*p* < 0.001).

The induction of COX-2 by *S. aureus* in HOK cells was found in our previous study [19], but that study did not fully reveal the functional mediator(s) exerting the inductive effect. Thus, using heat-inactivated *S. aureus* and supernatants from *S. aureus* cultures, we investigated whether the live cells or the secretory components of *S. aureus* were essential for the activation of the COX-2/PGE_2_ pathway in HOK cells. COX-2 expression in HOK cells cocultured with live or inactivated *S. aureus* was increased compared with that in untreated cells. Specifically, the COX-2 mRNA levels in HOK cells treated with live *S. aureus* and with heat-inactivated *S. aureus* were upregulated 1.43- and 1.35-fold, respectively, compared with those in the control cells, while the protein expression was also increased 1.13- and 1.29-fold, respectively, indicating an inductive effect of both live and inactivated *S. aureus* on COX-2 expression in HOK cells (Figure 1a-c). In contrast to the observations in *S. aureus*-treated HOK cells, no changes in COX-2 expression were found between the supernatant-treated HOK cells and the control cells.

We next evaluated PGE_2_ production by ELISA and found that HOK cells cocultured with live *S. aureus* and with heat-inactivated *S. aureus* produced PGE_2_ at a level of 502.16 pg/mL and 516.96 pg/mL, respectively, both of which were higher than the 383.12 pg/mL produced by untreated HOK cells. In contrast, cells treated with *S. aureus* culture supernatant produced a lower level of PGE_2_ than control cells. Using NS-398, a small molecule-specific inhibitor of COX-2, we then investigated whether the differences in PGE_2_ production were COX-2-induced. As shown in Figure 1d, after treatment with NS-398, all cells produced a similarly low level of PGE_2_, indicating that the inhibition of COX-2 markedly decreased PGE_2_ production and inhibited the promotive effect of *S. aureus* on PGE_2_ production. Taken together, these results demonstrated that the *S. aureus* cell rather than its secretory components functions to induce COX-2 expression and consequently to increase PGE_2_ production in HOK cells and that the live/dead status of *S. aureus* is not strongly essential for the activation of the COX-2/PGE_2_ pathway.

### S. aureus facilitates HOK cell proliferation in a PGE2-independent manner

**Figure 2.**
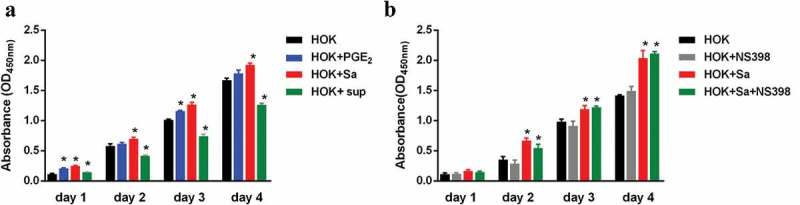
*S. aureus* facilitates HOK cell proliferation in a PGE2-independent manner. (a) Proliferation of HOK cells treated with PGE_2_, heat-inactivated *S. aureus* or *S. aureus* supernatants (n = 5). (b) Proliferation of HOK cells and *S. aureus*-infected HOK cells after treatment with NS-398, a small molecule specific inhibitor of COX-2 (n = 5). The data are presented as the means ± standard errors, and the asterisks represent significant differences (^＊^*p* < 0.05) compared with the non-treated group.

HOK cells were treated with PGE_2_, heat-inactivated bacterial cells or supernatants from *S. aureus* cultures, and cell proliferation was determined by a cell counting kit (CCK-8). The proliferation rate of HOK cells cocultured with *S. aureus* were significantly higher at all experimental time points than that of control cells (Figure 2(a)). A similar phenomenon was also observed in HOK cells treated with PGE_2_. However, the proliferation of supernatant-treated HOK cells was significantly decreased compared with that of control cells. These results indicated that both inactivated *S. aureus* and PGE_2_ can facilitate HOK cell proliferation, but the supernatants from *S. aureus* cultures cannot. To further investigate whether the promotive effect of *S. aureus* on HOK cell proliferation resulted from COX-2-derived PGE_2_, we used NS-398 to suppress PGE_2_ production and then evaluated the proliferation of HOK cells treated with *S. aureus*. The results shown in Figure 2(b) revealed that NS-398-treated HOK cells had proliferation rates similar to those of untreated cells. When stimulated with *S. aureus*, both untreated and NS-398-treated HOK cells exhibited a higher proliferation rate than the control. Thus, the promotive effect of *S. aureus* on HOK cell proliferation was not associated with NS-398. Taken together, these results demonstrated that *S. aureus* can facilitate the proliferation of HOK cells via a mechanism independent of PGE_2_.

### Upregulation of COX-2 determines S. aureus-induced HOK cell proliferation

**Figure 3.**
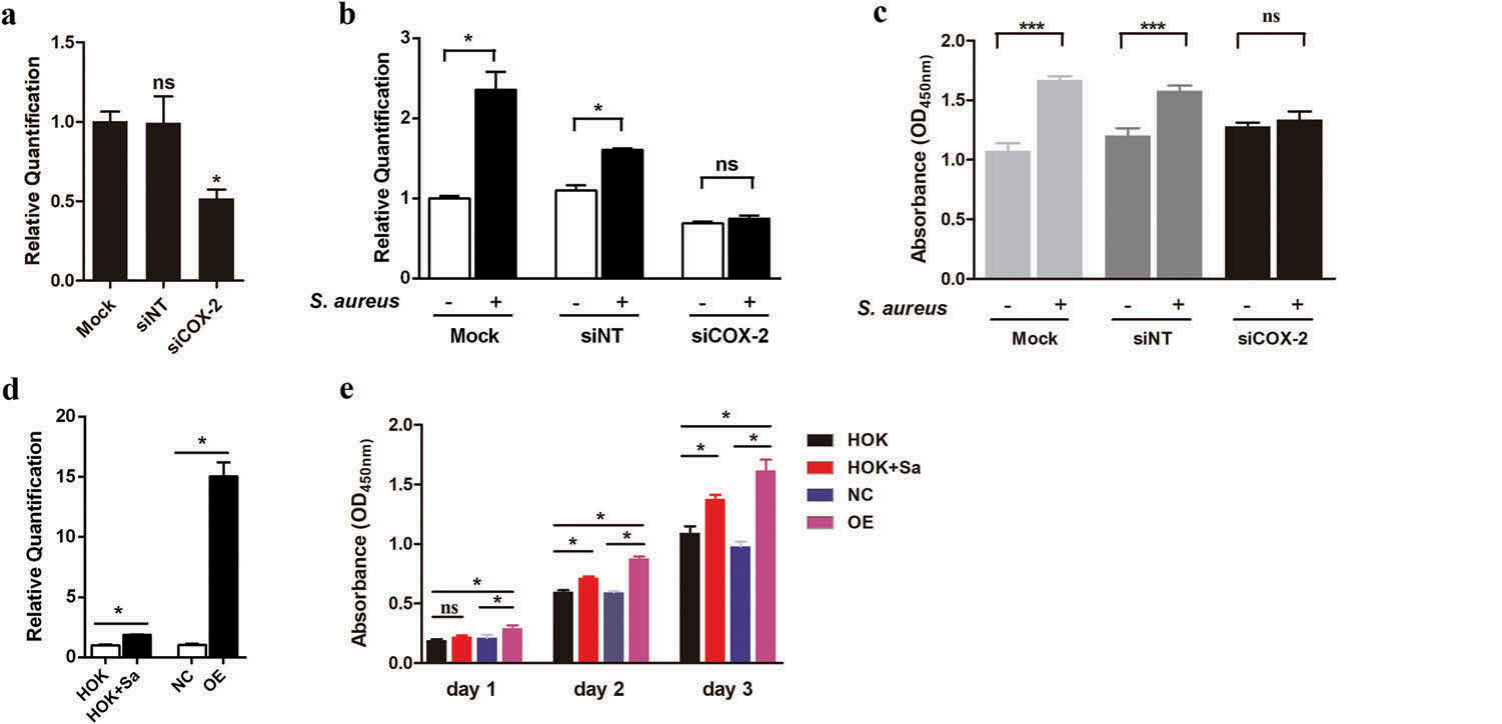
Upregulation of COX-2 contributes to the promotion of HOK cell proliferation. (a) COX-2 mRNA levels in mock-, negative control (siNT)- and siCOX-2-treated HOK cells (n = 3). (b) Expression of mCOX-2 in mock-, siNT- and siCOX-2-treated HOK cells after coculture with heat-inactivated *S. aureus* for 24 h (n = 3). (c) Proliferation of mock-treated cells, siNT-treated cells and siCOX-2-treated HOK cells after coculture with *S. aureus* (n = 6). (d) Expression of mCOX-2 in OE cells, NC cells, HOK cells and *S. aureus*-infected HOK cells (n = 3). (e) Proliferation of HOK cells infected with *S. aureus* and overexpressing COX-2 (n = 6). The data are presented as the means ± standard errors, and the asterisks represent significant differences (^＊^*p* < 0.05, ^＊＊^*p* < 0.01, ^＊＊＊^*p* < 0.001) between the compared groups.

Using small interfering RNA, we knocked down COX-2 mRNA in HOK cells and then evaluated HOK cell proliferation with the CCK-8 kit. The levels of COX-2 mRNA were quantified by qPCR to confirm the siRNA interference efficiency (Figure 3(a)). A lower level of COX-2 mRNA was observed in cells transfected with siCOX-2 than in cells transfected with the mock (control) or siNT (negative control). In addition, after coculture with heat-inactivated *S. aureus*, both the mock- and siNT-transfected cells displayed higher levels of COX-2 mRNA than the corresponding uninfected cells. However, no change in the COX-2 mRNA level was found in siCOX-2-transfected cells after *S. aureus* treatment, suggesting that *S. aureus* failed to induce COX-2 expression in siCOX-2-transfected cells during the experimental time period (Figure 3(b)). The CCK-8 assay revealed that *S. aureus* increased the proliferation of mock-transfected cells and siNT-transfected cells but had no effect on the proliferation of siCOX-2 cells (Figure 3(c)). Thus, upregulation of COX-2 is essential for the promotive effect of *S. aureus* on HOK proliferation.

To further confirm the hypothesis that upregulation of COX-2 promoted HOK cell proliferation, we constructed a COX-2 overexpression HOK cell line (OE cells) and assessed cell proliferation. The expression of mCOX-2 was much higher in OE cells than in NC cells and control HOK cells (Figure 3(d)). Cells transfected with the lentiviral vector CON238 were named NC cells and were the negative control. Notably, COX-2 mRNA expression in OE cells was markedly upregulated compared with that in *S. aureus*-treated HOK cells. In the CCK-8 assay, *S. aureus* was found to promote HOK proliferation at day 2 and day 3, while OE cells, at all the experimental time points, displayed a marked increase in proliferation compared with that of NC cells and control cells (Figure 3(e)). Taken together, upregulation of COX-2 in HOK cells can promote cell proliferation and *S. aureus* may facilitate HOK cell proliferation by upregulating COX-2 expression.

### *S. aureus regulated the transcription of* cyclin D1, p16 *and* Rb, *and promoted malignant transformation in HOK cells*

**Figure 4.**
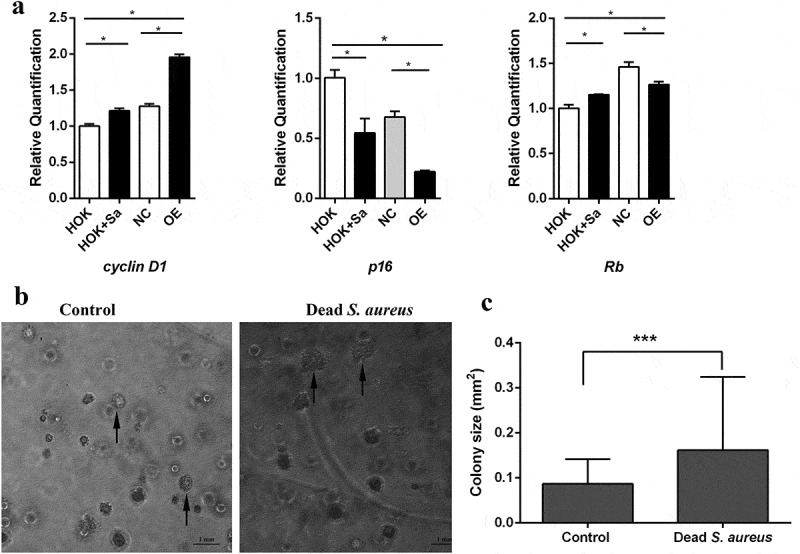
*S. aureus* regulated the transcription of *cyclin D1, p16* and *Rb*, and promoted malignant transformation in HOK cells. (a) mRNA expression levels of the *cyclin D1, p16* and *Rb* genes in OE cells, NC cells, HOK cells and *S. aureus*-infected HOK cells (n = 3). (b) Representative images of colony formation on soft agar. (c) Size of each colony in three random images. The data are presented as the means ± standard errors, and the asterisks represent significant differences (^＊^*p* < 0.05, ^＊＊^*p* < 0.01, ^＊＊＊^*p* < 0.001) between the compared groups.

Cell proliferation is consequently influenced by the cell cycle, which is regulated by many genes. Previous studies have shown that the genes *cyclin D1, p16* and *Rb* are commonly altered in many human malignancies. Thus, we next investigated whether the induction of COX-2 in HOK cells affected the expression of genes involved in the cell cycle. The results shown in Figure 4(a) revealed that *S. aureus*-infected HOK cells exhibited higher levels of *cyclin D1* mRNA and lower levels of *p16* mRNA than uninfected HOK cells. Meanwhile, OE cells displayed higher levels of *cyclin D1* mRNA and lower levels of *p16* and *Rb* than NC cells. These results indicated that COX-2 is involved in regulating genes associated with the cell cycle in HOK cells and that the change in the expression of these cell cycle genes is consistent with the mCOX-2 expression level. We also tested the potential for malignant transformation in HOK cells after infection with *S. aureus* by a soft agar colony formation assay. The images in Figure 4(b) show a larger colony in the cells treated with nonviable *S. aureus* compared with the non-treated control cells. The size of each colony in three random images was measured. The average size in the *S. aureus* group was larger than that in the control group (Figure 4(c)), indicating that *S. aureus* infection promoted colony formation and malignant transformation in HOK cells.

### RNA-sequence analysis identified critical pathways/genes regulated by COX-2 signaling

RNA sequencing of the six samples (triplicates of NC cells and triplicates of OE cells) was performed. The annotation results revealed that a total of 3,428 unigenes were classified into 26 EggNOG categories, among which the most enriched were ‘Function unknown’ and ‘General function prediction only’, followed by ‘Signal transduction mechanisms’ and ‘Posttranslational modification, protein turnover, chaperones’ (Supplementary Figure 1).

**Figure 5.**
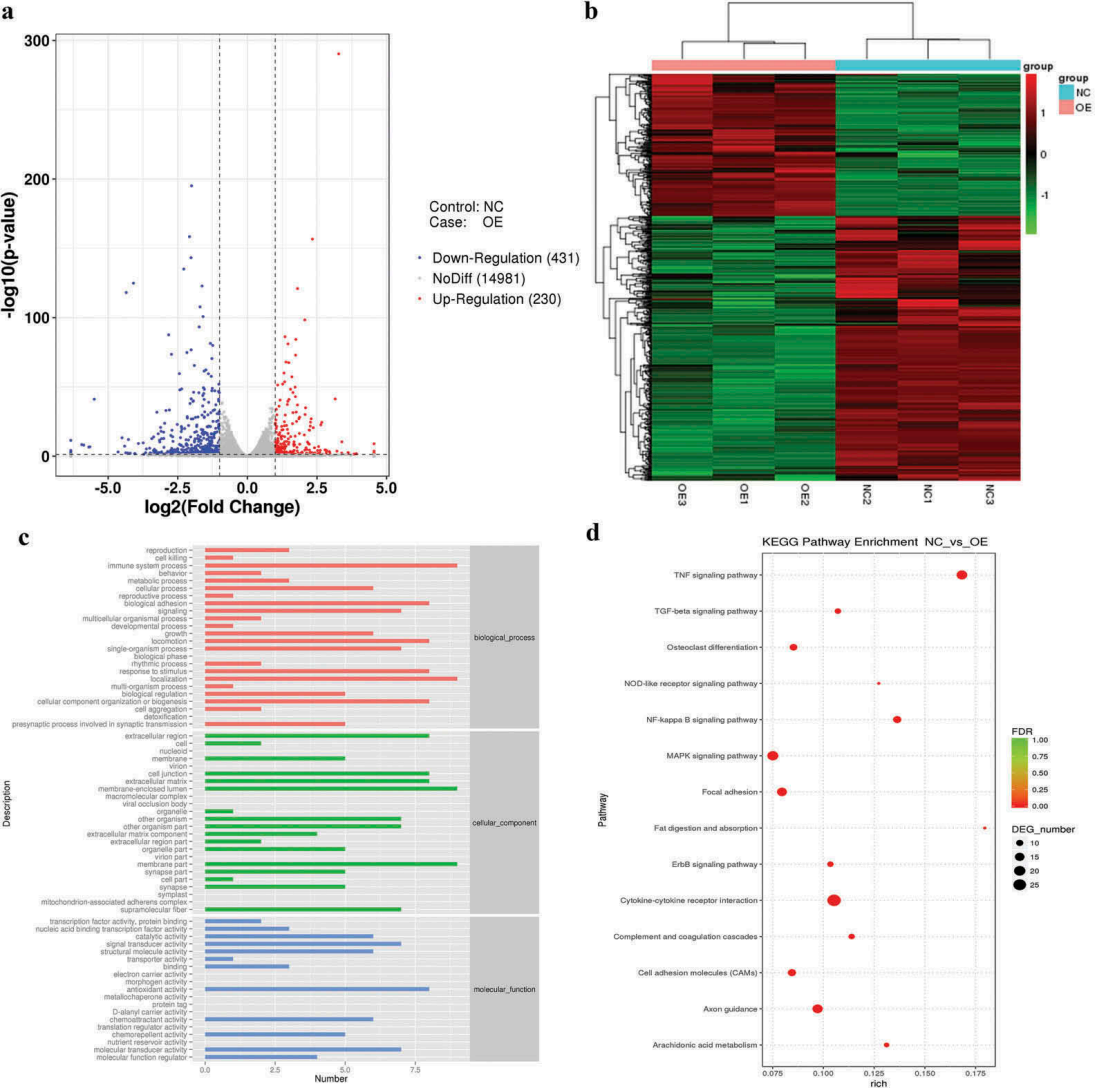
Transcriptome analysis of the RNA-sequence data for OE and NC cells. (a) Volcano plot indicating DEGs between OE and NC cells. (b) Hierarchical cluster analysis of DEGs. The heat map represents the cluster analysis of 661 DEGs according to the gene expression level. Expression levels were measured as RPKM-normalized values. Each row represents a differentially expressed gene, while each column represents a sample. The green and red colour gradients indicate decreases and increases in transcript abundances, respectively. (c) GO category distribution of the 661 DEGs among level 1 GO categories: biological process (BP), molecular function (MF), and cellular component (CC). A *p*-value and a false discovery rate (FDR) are given for significance. (d) The top 14 most significantly enriched pathways from the KEGG pathway analysis of differentially expressed genes. The pathways shown were identified at a threshold FDR of < 5%. The size of the dots represents the number of DEGs enriched in the pathway. The larger the dot is, the more DEGs are enriched . The term ‘richness factor’ means the ratio of the number of DEGs to the number of genes annotated in a particular pathway. The greater the richness factor, the greater the degree of enrichment.

Differentially expressed genes between OE and NC cells were identified to reveal the regulatory effect of COX-2 on the transcriptome of HOK cells. In total, 661 DEGs were obtained at the selected cutoff thresholds, namely, | fold change | ≥ 2 and *p* < 0.05. Among the DEGs, 230 were upregulated, while 431 were downregulated in OE cells compared to their expression in NC cells (Figure 5(a)). Hierarchical cluster analysis of the 661 DEGs was performed using normalized read counts to investigate gene expression dynamics in the six samples. The clustering analysis results shown in Figure 5(b) revealed that the gene signature of OE cells was distinct from that of NC cells, suggesting significant changes in the transcriptome of HOK cells due to COX-2 overexpression.

Gene Ontology (GO) enrichment and KEGG pathway analysis were performed to better understand the functions of DEGs in the regulation of COX-2 in HOK cells and to further explore the possible mechanism underlying the regulation. As shown in Figure 5(c), the groups ‘immune system process’, ‘localization’, ‘membrane-enclosed lumen’, ‘membrane part’, and ‘antioxidant activity’, were the most highly represented.

The KEGG analysis showed that the 661 DEGs in OE and NC cells were significantly enriched in 44 pathways. Of these pathways, 14 under the threshold of FDR < 5% were identified. The ‘Cytokine-cytokine receptor interaction’ pathway had the highest number of enriched DEGs, followed by the ‘MAPK signaling pathway’, the ‘TNF signaling pathway’, the ‘Axon guidance’ pathway, the ‘Focal adhesion’ pathway and the ‘NF-kappa B signaling pathway’. Moreover, when a richness factor (the ratio of the number of DEGs to the number of genes annotated in a particular pathway) was incorporated as a filter, the ‘Fat digestion and absorption’ pathway, the ‘TNF signaling pathway’ and the ‘NF-kappa B signaling pathway’ had the highest degree of enrichment (Figure 5(d)). Of all the pathways, the ‘TNF signaling pathway’ as well as the ‘NF-kappa B signaling pathway’ and the ‘Cytokine-cytokine receptor interaction’ pathway were the most significantly disturbed by COX-2 overexpression, having both higher numbers of enriched DEGs and higher richness factors than the other identified pathways.

**Figure 6.**
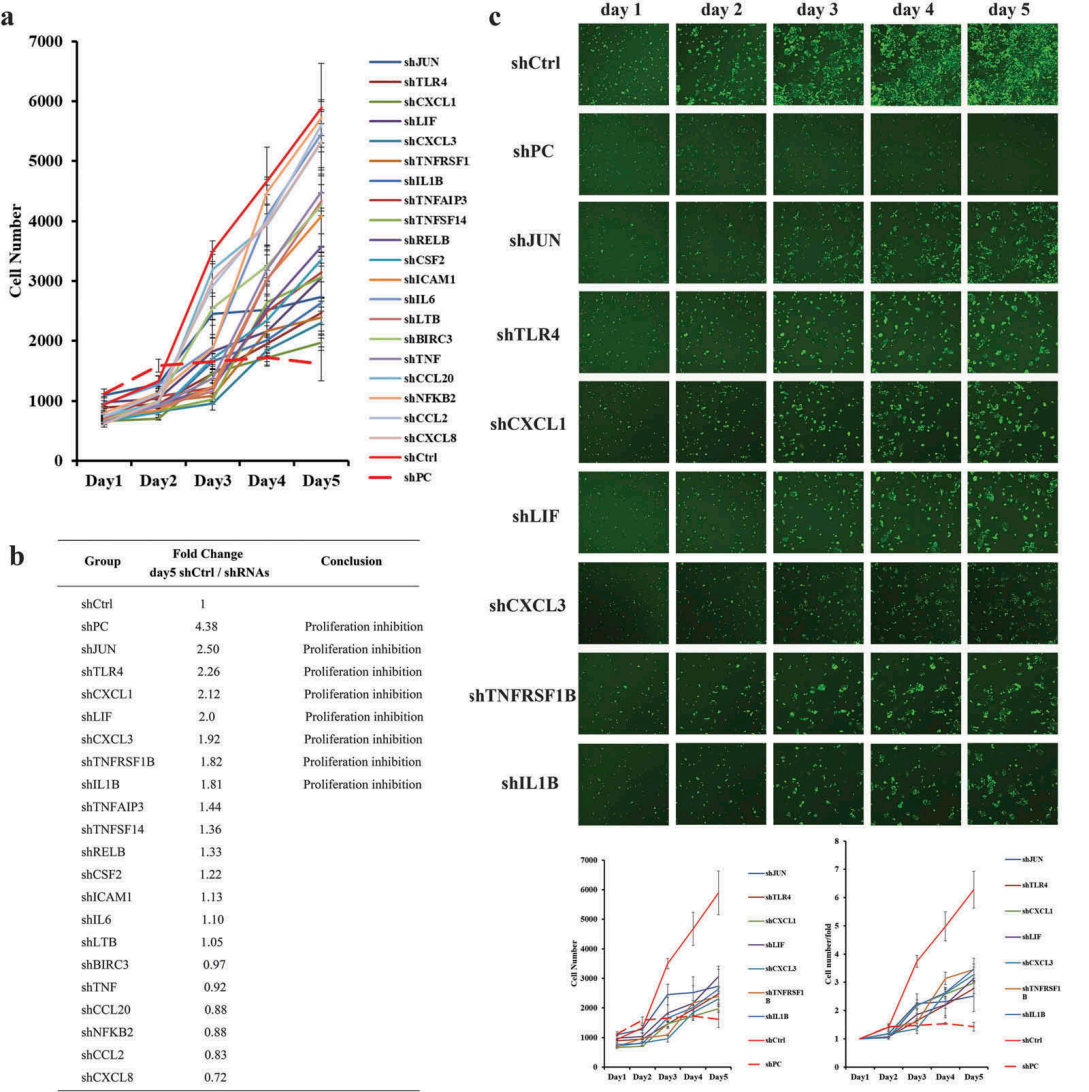
Functional gene analysis by the high-content screening (HCS) assay. (a) Proliferation of HOK cells treated with different shRNAs. shCtrl: Non-targeting shRNA (negative control). shPC: Proto-oncogene specific-targeting shRNA (positive control). (b) Fold change in the proliferation of HOK cells treated with different shRNAs on the fifth day after treatment. The fold change was calculated by the ratio of shCtrl (Day 5:Day 1)/the ratio of shRNAs (Day 5:Day 1). (c) Proliferation inhibition of HOK cells treated with shRNAs (shJUN, shTLR4, shCXCL1, shLIF, shCXCL3, shTNFRSF1β, or shIL-1β).

High-content screening (HCS) of the cell proliferation assay was performed to detect the functional genes in the ‘NF-kappa B signaling pathway’ and ‘TNF signaling pathway’. Twenty upregulated genes with the largest fold change in the two pathways were selected, and HOK cells were transfected with shRNAs targeting these 20 genes. Cell proliferation was inhibited to different degrees in cells transfected with different lentiviral shRNAs (Figure 6(a)). The cell number fold change (shCtrl/shRNAs) on the fifth day was calculated. Compared with the non-targeting shRNA (shCtrl) group, seven shRNA groups (shJUN, shTLR4, shCXCL1, shLIF, shCXCL3, shTNFRSF1β, and shIL-1β) with the largest fold change showed appreciable proliferation inhibition. The integrated green fluorescence intensity in these seven shRNA groups was much weaker than that in the shCtrl group (Figure 6(c)). Proliferation in the shJUN, shTLR4, shCXCL1, shLIF, shCXCL3, shTNFRSF1β, and shIL-1β groups was inhibited 2.50-, 2.26-, 2.12-, 2.0-, 1.92-, 1.82-, and 1.81-fold, respectively (Figure 6(b)).

## Discussion

It has been suggested that COX-2 promotes oral epithelial carcinogenesis and carcinoma malignancy through increased PGE_2_ production [40]. In cells from other tracts, COLO-320DM cells from the intestinal tract, for example, overexpression of COX-2 also resulted in a PGE_2_-dependent increase in the growth rate [41]. In the present study, however, we found that *S. aureus* facilitated HOK cell proliferation by upregulating COX-2 expression independent of PGE_2_ production. This inconsistency may be due to the complexity of the mechanisms by which COX-2/PGE_2_ affects cell proliferation. Additionally, there may be other mechanisms independent of the COX-2/PGE_2_ pathway or dependent on COX-2 at the transcriptional level for the promotive effect of *S. aureus* on HOK cell proliferation.

The *Rb, cyclin D1* and *p16* genes have been shown to be commonly altered in human malignancies [42–45]. Retinoblastoma protein (pRB), coded by the retinoblastoma (*Rb*) gene, is one of the most important components of the G1 checkpoint and has been suggested to play an important role in controlling cellular growth [46]. The activity of pRB is regulated by its phosphorylation, which is mediated by genes such as *cyclin D1* and *p16*. Ectopic expression of *cyclin D1* accelerates the G1/S phase transition, resulting in increases in genomic instability and the risk of carcinogenesis [42,43]. *p16*, also known as multiple tumor suppressor gene 1 (multiple tumor suppressor gene 1, MTS1), is a cyclin-dependent kinase inhibitor (CDKI) that inhibits Cyclin D1-CDK4/6 complexes, and its inactivation has been observed in head and neck cancer [44]. In addition, it has been reported that more than 90% of oral tumours have at least one abnormality affecting either retinoblastoma, *cyclin D1* or *p16* [45,47]. In the present study, we demonstrated that overexpression of COX-2 in OE cells upregulated *cyclin D1* and downregulated *p16* expression, while high expression of COX-2 in HOK cells induced by *S. aureus* infection upregulated *cyclin D1*, and downregulated *p16*. This finding confirms the observations in the proliferation assay, which showed that the cell line with COX-2 overexpression displayed an increase in proliferation compared to that of the infected cells. These results suggested that upregulation of COX-2 in HOK cells affected the expression of *cyclin D1* and *p16*, which might subsequently influence cell proliferation.

It has been noted that infections inducing cell proliferation might increase the incidence of cell transformation and the rate of tumour development [31]. Since we demonstrated that *S. aureus* infection promoted cell proliferation and regulated the expression of cell cycle genes in HOK cells, the question of whether infection could promote cancer transition needed to be solved. Therefore, we performed a soft agar colony formation assay, a technique widely used to evaluate the malignant transformation of cells [48]. The larger colonies of infected cells proved the potential of *S. aureus* to induce infection-associated malignant transformation.

According to the KEGG pathway analysis, the ‘TNF signaling pathway’ and the ‘NF−kappa B signaling pathway’ displayed a significant response to COX-2 overexpression, having higher numbers of enriched DEGs and higher richness factors. TNF-α, despite its ability to induce tumour necrosis, is thought to be another proinflammatory cytokine involved in cancer, and there is ample evidence that TNF-α acts as a tumour promoter in several experimental models of cancer [49,50]. Various members of the TNF superfamily have been shown to be involved in the proliferation and survival of cells, commonly through a cell signaling pathway that mediates the activation of NF-kappa B (NF-κB) and mitogen-activated protein kinases [51–53]. NF-κB is an inducible transcription factor mediating signal transduction between the cytoplasm and nucleus and is thought to be the linchpin in inflammation-associated cancer [54]. NF-κB is a major activator of anti-apoptotic gene expression and has been shown to play a role in cell proliferation, transformation, and tumour development [55–57]. In addition, it has been reported that most cytokines act through NF-κB signaling, which induces the expression of a wide spectrum of cytokines, forming a complex, crosstalking network. Thus, the significant response of the ‘NF−kappa B signaling pathway’ and ‘TNF signaling pathway’ to COX-2 overexpression in our present study suggested the potential of *S. aureus* to induce malignant transformation in HOK cells.

To explore the functional genes in the ‘NF−kappa B signaling pathway’ and ‘TNF signaling pathway’ responsible for the promotive effect of COX-2 on HOK cell proliferation, an RNA interference assay based on HCS was performed. Silencing the genes *jun, tlr4, cxcl1, lif, cxcl3, tnfrsf1β*, and *il1β* inhibited cell proliferation, indicating that these seven genes are critical for cell proliferation and might be associated with the function of COX-2. However, the correlation between the genes *jun, tlr4, cxcl1, lif, cxcl3, tnfrsf1β*, and *il1β* and COX-2 needs further exploration and verification.

## Conclusion

The COX-2/PGE_2_ axis can be another mechanism underlying inflammation-associated cancer. A sufficient understanding of the role of COX-2/PGE_2_ in inflammation and cancer is beneficial to cancer prevention and treatment. In our present study, we revealed an interesting phenomenon that overexpression of COX-2 by *S. aureus* infection facilitates HOK proliferation independent of PGE_2_ production and regulates the oral cancer-associated genes *cyclin D1* and *p16*. In addition, *S. aureus* infection promotes malignant transformation in HOK cells. The ‘NF−kappa B signaling pathway’ and ‘TNF signaling pathway’ may form candidate mechanisms for the promotive effect of COX-2 on HOK cell proliferation. Seven upregulated genes (*jun, tlr4, cxcl1, lif, cxcl3, tnfrsf1β*, and *il1β*) in these two pathways were critical for the increased proliferation rate of HOK cells and might be associated with the function of COX-2.

## References

[1] Kim G-Y, Lee CH. Antimicrobial susceptibility and pathogenic genes of *Staphylococcus aureus* isolated from the oral cavity of patients with periodontitis. J Periodontal Implant Sci. 2015;45(6):223–12. 26734493 10.5051/jpis.2015.45.6.223 PMC4698949

[2] McCormack MG, Smith AJ, Akram AN, et al *Staphylococcus aureus* and the oral cavity: an overlooked source of carriage and infection? Am J Infect Control. 2015;43(1):35–37. 25564121 10.1016/j.ajic.2014.09.015

[3] Ohara-Nemoto Y, Haraga H, Kimura S, et al Occurrence of staphylococci in the oral cavities of healthy adults and nasal-oral trafficking of the bacteria. J Med Microbiol. 2008;57(Pt1):95–99. 18065673 10.1099/jmm.0.47561-0

[4] Kronstrom M, Svenson B, Hellman M, et al Early implant failures in patients treated with Branemark system titanium dental implants: A retrospective study. Int J Oral Maxillofac Implants. 2001;16(2):201–207. 11324208

[5] Rokadiya S, Malden NJ An implant periapical lesion leading to acute osteomyelitis with isolation of *Staphylococcus aureus*. Br Dent J. 2008;205(9):489–491. 18997702 10.1038/sj.bdj.2008.935

[6] Dancer SJ The effect of antibiotics on methicillin-resistant *Staphylococcus aureus*. J Antimicrob Chemother. 2008;61(2):246–253. 18057071 10.1093/jac/dkm465

[7] Ippolito G, Leone S, Lauria FN, et al Methicillin-resistant *Staphylococcus aureus*: the superbug. Int J Infect Dis. 2010;14(Suppl 4):S7–S11. 20851011 10.1016/j.ijid.2010.05.003

[8] Scully C, Bagan JV Oral squamous cell carcinoma: overview of current understanding of aetiopathogenesis and clinical implications. Oral Dis. 2009;15(6):388–399. 19371401 10.1111/j.1601-0825.2009.01563.x

[9] Mathews J, Patel M Bacterial endotoxins and microorganisms in the oral cavities of patients on cancer therapy. Microb Pathog. 2018;123:190–195. 30016679 10.1016/j.micpath.2018.07.014

[10] Almstahl A, Finizia C, Carlen A, et al Mucosal microflora in head and neck cancer patients. Int J Dent Hyg. 2018;16(4):459–466. 29766652 10.1111/idh.12348

[11] Fang Y, Chen X, Bajpai M, et al Cellular origins and molecular mechanisms of Barrett’s esophagus and esophageal adenocarcinoma. Ann N Y Acad Sci. 2013;1300:187–199. 24117642 10.1111/nyas.12249

[12] Scarpa M, Castagliuolo I, Castoro C, et al Inflammatory colonic carcinogenesis: A review on pathogenesis and immunosurveillance mechanisms in ulcerative colitis. World J Gastroenterol. 2014;20(22):6774–6785. 24944468 10.3748/wjg.v20.i22.6774 PMC4051917

[13] Moore PS, Chang Y Why do viruses cause cancer? Highlights of the first century of human tumour virology. Nat Rev Cancer. 2010;10(12):878–889. 21102637 10.1038/nrc2961 PMC3718018

[14] Elinav E, Nowarski R, Thaiss CA, et al Inflammation-induced cancer: crosstalk between tumours, immune cells and microorganisms. Nat Rev Cancer. 2013;13(11):759–771. 24154716 10.1038/nrc3611

[15] Moet GJ, Jones RN, Biedenbach DJ, et al Contemporary causes of skin and soft tissue infections in North America, Latin America, and Europe: report from the SENTRY antimicrobial surveillance program (1998–2004). Diagn Microbiol Infect Dis. 2007;57(1):7–13. 17059876 10.1016/j.diagmicrobio.2006.05.009

[16] Farnsworth CW, Schott EM, Benvie AM, et al Obesity/type 2 diabetes increases inflammation, periosteal reactive bone formation, and osteolysis during *Staphylococcus aureus* implant-associated bone infection. J Orthop Res. 2018;36(6):1614–1623. 29227579 10.1002/jor.23831 PMC5995608

[17] Diallo K, Thilly N, Luc A, et al Management of bloodstream infections by infection specialists: an international ESCMID cross-sectional survey. Int J Antimicrob Agents. 2018;51(5):794–798. 29309899 10.1016/j.ijantimicag.2017.12.010

[18] Bettenworth D, Nowacki TM, Friedrich A, et al Crohn’s disease complicated by intestinal infection with methicillin-resistant *Staphylococcus aureus*. World J Gastroenterol. 2013;19(27):4418–4421. 23885156 10.3748/wjg.v19.i27.4418 PMC3718913

[19] Wang Y, Ren B, Zhou X, et al Growth and adherence of *Staphylococcus aureus* were enhanced through the PGE(2) produced by the activated COX-2/PGE(2) pathway of infected oral epithelial cells. PLos One. 2017;12(5):e0177166. 28472126 10.1371/journal.pone.0177166 PMC5417706

[20] Park JY, Pillinger MH, Abramson SB Prostaglandin E-2 synthesis and secretion: the role of PGE(2) synthases. Clin Immunol. 2006;119(3):229–240. 16540375 10.1016/j.clim.2006.01.016

[21] Jain S, Chakraborty G, Raja R, et al Prostaglandin E-2 regulates tumor angiogenesis in prostate cancer. Cancer Res. 2008;68(19):7750–7759. 18829529 10.1158/0008-5472.CAN-07-6689

[22] Sheng HM, Shao JY, Washington MK, et al Prostaglandin E-2 increases growth and motility of colorectal carcinoma cells. J Biol Chem. 2001;276(21):18075–18081. 11278548 10.1074/jbc.M009689200

[23] Greenhough A, Smartt HJM, Moore AE, et al The COX-2/PGE(2) pathway: key roles in the hallmarks of cancer and adaptation to the tumour microenvironment. Carcinogenesis. 2009;30(3):377–386. 19136477 10.1093/carcin/bgp014

[24] Santoro A, Bufo P, Russo G, et al Expression and clinical implication of cyclooxygenase-2 and e-cadherin in oral squamous cell carcinomas. Cancer Biol Ther. 2015. 10.1080/15384047.2015.1071741 PMC7537792 26218314

[25] Kapoor V, Singh AK, Dey S, et al Circulating cycloxygenase-2 in patients with tobacco-related intraoral squamous cell carcinoma and evaluation of its peptide inhibitors as potential antitumor agent. J Cancer Res Clin Oncol. 2010;136(12):1795–1804. 20213098 10.1007/s00432-010-0837-4 PMC11827827

[26] Morita Y, Morita N, Hata K, et al Cyclooxygenase-2 expression is associated with vascular endothelial growth factor-c and lymph node metastasis in human oral tongue cancer. Oral Surg Oral Med Oral Pathol Oral Radiol. 2014;117(4):502–510. 24560404 10.1016/j.oooo.2013.12.410

[27] Shibata M, Kodani I, Osalki M, et al Cyclo-oxygenase-1 and-2 expression in human oral mucosa, dysplasias and squamous cell carcinomas and their pathological significance. Oral Oncol. 2005;41(3):304–312. 15743693 10.1016/j.oraloncology.2004.09.009

[28] Yu HP, Xu SQ, Liu L, et al Cyclooxygenase-2 expression in squamous dysplasia and squamous cell carcinoma of the esophagus. Cancer Lett. 2003;198(2):193–201. 12957358 10.1016/s0304-3835(03)00340-9

[29] Yang S-F, Chen M-K, Hsieh Y-S, et al Prostaglandin E-2/EP1 signaling pathway enhances intercellular adhesion molecule 1 (ICAM-1) expression and cell motility in oral cancer cells. J Biol Chem. 2010;285(39):29808–29816. 20647315 10.1074/jbc.M110.108183 PMC2943269

[30] Divvela AKC, Challa SR, Tagaram IK Pathogenic role of cyclooxygenase-2 in cancer. J Health Sci. 2010;56(5):502–516.

[31] Lax AJ, Thomas W How bacteria could cause cancer: one step at a time. Trends Microbiol. 2002;10(6):293–299. 12088666 10.1016/s0966-842x(02)02360-0

[32] Takagi M, Yamamoto D, Ogawa S, et al Messenger RNA expression of anglotensin-converting enzyme, endothelin, cyclooxygenase-2 and prostaglandin synthases in bovine placentomes during gestation and the postpartum period. Vet J. 2008;177(3):398–404. 17804266 10.1016/j.tvjl.2007.05.017

[33] Zbinden C, Stephan R, Johler S, et al The inflammatory response of primary bovine mammary epithelial cells to *Staphylococcus aureus* strains is linked to the bacterial phenotype. PLos One. 2014;9(1):e87374. 24498088 10.1371/journal.pone.0087374 PMC3907564

[34] Chen JH, Perry CJ, Tsui Y-C, et al Prostaglandin E2 and programmed cell death 1 signaling coordinately impair CTL function and survival during chronic viral infection. Nat Med. 2015;21(2):327–334. 25799228 10.1038/nm.3831 PMC4505619

[35] Shi J, Ma C, Qi D, et al Transcriptional responses and flavor volatiles biosynthesis in methyl jasmonate-treated tea leaves. BMC Plant Biol. 2015;15:233. 26420557 10.1186/s12870-015-0609-z PMC4588909

[36] Wu Z, Liu Y, Dong W, et al CD14 in the TLRs signaling pathway is associated with the resistance to *E. coli* F18 in Chinese domestic weaned piglets. Sci Rep. 2016;6:24611. 27098998 10.1038/srep24611 PMC4838916

[37] Langmead B, Salzberg SL Fast gapped-read alignment with Bowtie 2. Nat Methods. 2012;9(4):357–359. 22388286 10.1038/nmeth.1923 PMC3322381

[38] Kim D, Pertea G, Trapnell C, et al TopHat2: accurate alignment of transcriptomes in the presence of insertions, deletions and gene fusions. Genome Biol. 2013;14(4):R36. 23618408 10.1186/gb-2013-14-4-r36 PMC4053844

[39] Moffat J, Grueneberg DA, Yang XP, et al A lentiviral RNAi library for human and mouse genes applied to an arrayed viral high-content screen. Cell. 2006;124(6):1283–1298. 16564017 10.1016/j.cell.2006.01.040

[40] Husvik C, Khuu C, Bryne M, et al PGE(2) Production in oral cancer cell lines is COX-2-dependent. J Dent Res. 2009;88(2):164–169. 19278989 10.1177/0022034508329519

[41] Kinoshita T, Takahashi Y, Sakashita T, et al Growth stimulation and induction of epidermal growth factor receptor by overexpression of cyclooxygenases 1 and 2 in human colon carcinoma cells. Biochim Biophys Acta. 1999;1438(1):120–130. 10216286 10.1016/s1388-1981(99)00034-7

[42] Dhingra V, Verma J, Misra V, et al Evaluation of cyclin D1 expression in head and neck squamous cell carcinoma. J Clin Diagn Res. 2017;11(2):EC1–EC4. 10.7860/JCDR/2017/21760.9329 PMC5376904 28384866

[43] Jiang W, Kahn SM, Zhou P, et al Overexpression of cyclin D1 in rat fibroblasts cause abnormalities in growth-control, cell-cycle progression and gene-expression. Oncogene. 1993;8(12):3447–3457. 8247550

[44] Reed AL, Califano J, Cairns P, et al High frequency of p16 (CDKN2/MTS-1/INK4A) inactivation in head and neck squamous cell carcinoma. Cancer Res. 1996;56(16):3630–3633. 8705996

[45] Nakahara Y, Shintani S, Mihara M, et al Alterations of Rb, p16(INK4A) and cyclin D1 in the tumorigenesis of oral squamous cell carcinomas. Cancer Lett. 2000;160(1):3–8. 11098077 10.1016/s0304-3835(00)00546-2

[46] Weinberg RA The retinoblastoma protein and cell-cycle control. Cell. 1995;81(3):323–330. 7736585 10.1016/0092-8674(95)90385-2

[47] Todd R, Hinds PW, Munger K, et al Cell cycle dysregulation in oral cancer. Crit Rev Oral Biol Med. 2002;13(1):51–61. 12097237 10.1177/154411130201300106

[48] Borowicz S, Van Scoyk M, Avasarala S, et al The soft agar colony formation assay. J Vis Exp. 2014;92:e51998. 10.3791/51998 PMC4353381 25408172

[49] Arnott CH, Scott KA, Moore RJ, et al Tumour necrosis factor-alpha mediates tumour promotion via a PKC alpha- and AP-1-dependent pathway. Oncogene. 2002;21(31):4728–4738. 12101411 10.1038/sj.onc.1205588

[50] Lind MH, Rozell B, Wallin RPA, et al Tumor necrosis factor receptor 1-mediated signaling is required for skin cancer development induced by NF-kappa B inhibition. Proc Natl Acad Sci U S A. 2004;101(14):4972–4977. 15044707 10.1073/pnas.0307106101 PMC387358

[51] Tracey KJ, Cerami A Tumor-necrosis-factor, other cytokines and disease. Annu Rev Cell Biol. 1993;9:317–343. 8280464 10.1146/annurev.cb.09.110193.001533

[52] Smith CA, Farrah T, Goodwin RG The TNF receptor superfamily of cellular and viral-proteins-activation, costimulation, and death. Cell. 1994;76(6):959–962. 8137429 10.1016/0092-8674(94)90372-7

[53] Liu ZG, Han J Cellular responses to tumor necrosis factor. Curr Issues Mol Biol. 2001;3(4):79–90. 11719971

[54] Pikarsky E, Porat RM, Stein I, et al NF-kappa B functions as a tumour promoter in inflammation-associated cancer. Nature. 2004;431(7007):461–466. 15329734 10.1038/nature02924

[55] Baeuerle PA, Henkel T Function and activation of NF-kappa-B in the immune-system. Annu Rev Immunol. 1994;12:141–179. 8011280 10.1146/annurev.iy.12.040194.001041

[56] VanAntwerp DJ, Martin SJ, Kafri T, et al Suppression of TNF-alpha-induced apoptosis by NF-kappa B. Science. 1996;274(5288):787–789. 8864120 10.1126/science.274.5288.787

[57] Beg AA, Baltimore D An essential role for NF-kappa B in preventing TNF-alpha-induced cell death. Science. 1996;274(5288):782–784. 8864118 10.1126/science.274.5288.782

