# Supplementary material for: Staphylococcus aureus induces COX-2-dependent proliferation and malignant transformation in oral keratinocytes
Source: J Oral Microbiol. 2019 Jul 22;11(1):1643205. doi: 10.1080/20002297.2019.1643205 (PMC6691923; doi:10.1080/20002297.2019.1643205)
Supplement: Supplemental Material [file ZJOM_A_1643205_SM2605.docx]

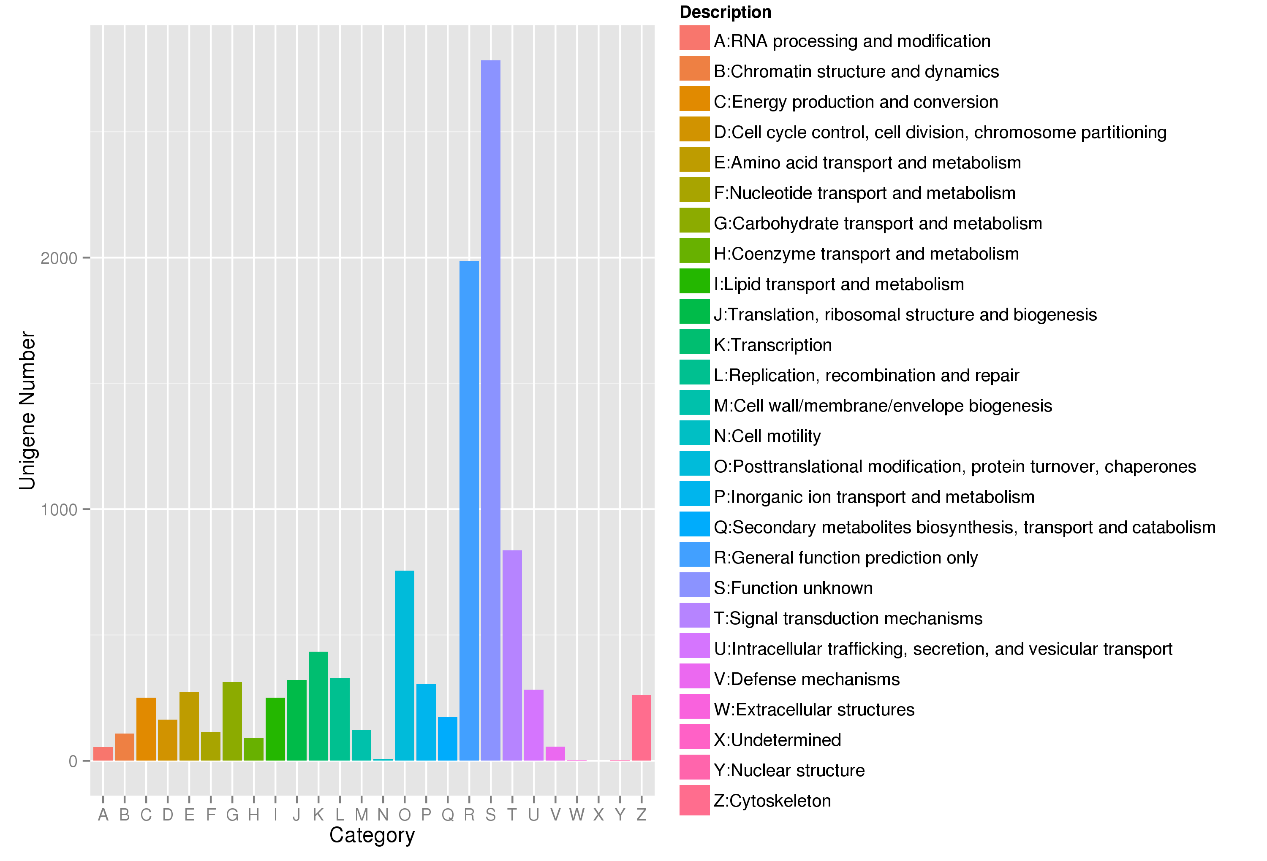


**Figure S1** Evolutionary genealogy of genes: Non-supervised Orthologous Groups (eggNOG) analysis. A total of 3428 unigenes were analyzed using eggnog database and subdivided into 26 clusters of orthologous (COG) classifications. x-axis, COG classifications; y-axis, number of unigenes.

**Table S1. Primers used in this study**

| Genes | PCR primers | |  |
| --- | --- | --- | --- |
| *cox-2* | | For 5'- CTCCTGTGCCTGATGATTGC -3' |  |
|  | | Rev 5'- CAGCCCGTTGGTGAAAGC -3' |  |
| *Cyclin D1* | | For 5'-TCTACACCGACAACTCCATCC-3' |  |
|  | | Rev 5'- TTCCACTTGAGCTTGTTCACC-3' |  |
| *Rb* | | For 5'- CCAGCACACCCTGCAGAAT-3' |  |
|  | | Rev 5'- TGCCATACATGGAACACATCATAA-3' |  |
| *P16* | | For 5'- CCCCTTGCCTGGAAAGATAC-3' |  |
|  | | Rev 5'- AGCCCCTCCTCTTTCTTCCT-3' |  |
| GAPDH | | For 5'-GTCTTCACTACCATGGAGAAGG-3' |  |
|  | | Rev 5'-TCATGGATGACCTTGGCCAG-3' |  |
